# Prevalence of Low Energy Availability in Collegiate Women Soccer Athletes

**DOI:** 10.3390/jfmk5040096

**Published:** 2020-12-18

**Authors:** Meghan K. Magee, Brittanie L. Lockard, Hannah A. Zabriskie, Alexis Q. Schaefer, Joel A. Luedke, Jacob L. Erickson, Margaret T. Jones, Andrew R. Jagim

**Affiliations:** 1 School of Kinesiology, George Mason University, Manassas, VA 20109, USA; mmagee2@gmu.edu; 2 Patriot Performance Laboratory, Frank Pettrone Center for Sports Performance, Intercollegiate Athletics, George Mason University, Fairfax, VA 22030, USA; blockard@gmu.edu (B.L.L.); jagim.andrew@mayo.edu (A.R.J.); 3 Division of Human Performance, University of the Incarnate Word, San Antonio, TX 78209, USA; 4 Department of Kinesiology, Towson University, Towson, MD 21252 USA; hzabriskie@towson.edu; 5 Athletics Department, University of Wisconsin—La Crosse, La Crosse, WI 54601, USA; aschaefer@uwlax.edu (A.Q.S.); jluedke@uwlax.edu (J.A.L.); 6 Sports Medicine, Mayo Clinic Health System, Onalaska, WI 54650, USA; erickson.jacob@mayo.edu

**Keywords:** energy availability, relative energy deficiency in sport, sports nutrition, women soccer athletes, nutrition knowledge, LEAF-Q

## Abstract

(1) **Background:** Limited information exists on the prevalence of low energy availability (LEA) in collegiate team sports. The purpose of this study was to examine the prevalence of LEA in collegiate women soccer players. (2) **Methods:** Collegiate women soccer athletes (*n* = 18, height: 1.67 ± 0.05 m; body mass: 65.3 ± 7.9 kg; body fat %: 24.9 ± 5.6%) had their body composition and sport nutrition knowledge assessed in the pre-season. Energy availability was assessed mid-season using a 4-day dietary log and activity energy expenditure values from a team-based monitoring system. A validated screening tool was used to screen for LEA. (3) **Results:** The screening tool classified 56.3% of athletes as at risk of LEA (<30 kcal/kg of FFM); however, the actual dietary intake identified 67% as LEA. Athletes identified as non-LEA consumed significantly more absolute (*p* = 0.040) and relative (*p* = 0.004) energy than LEA athletes. (4) **Conclusions:** There was a high prevalence of LEA among collegiate women soccer athletes. Although previously validated in women endurance athletes, the LEA screening tool was not effective in identifying those at risk of LEA in this sample of athletes.

## 1. Introduction

Athletes have specific dietary requirements in order to meet training demands and optimize performance [1]. Typically, athletes have higher activity levels; greater lean body mass; and require higher amounts of energy, protein, and carbohydrates per day compared to non-athletes. However, previous research has indicated that athletes often do not meet the nutritional guidelines specific to their level of training, with women athletes tending to exhibit dietary deficiencies more frequently than male athletes [2,3,4,5,6,7,8,9,10,11,12]. Insufficient energy intake may predispose an athlete to low energy availability (LEA), which is thought to be a primary contributor to a complex condition characterized by a multifactorial state of physiological dysfunction referred to as Relative Energy Deficiency in Sport (RED-S) syndrome [13]. The International Olympic Committee published a consensus statement on RED-S [13] which described RED-S as similar to the well-known Female Athlete Triad paradigm, but encompassing a broader definition to include a spectrum of physiological dysfunction attributable to chronic energy deficiency. This deficiency may result from insufficient energy intake, excessive energy expenditure from training, or a combination of both. Further, RED-S is commonly associated with reductions in performance, with a concomitant increased risk of health complications including, but not limited to, impairments in metabolism, menstrual function, bone health, immunity, protein synthesis, reproduction abilities, and cardiovascular health [13].

A common strategy to determine an athlete’s risk of energy deficiency is to assess energy availability level (kcal/kg FFM/day), which is calculated by subtracting the activity energy expenditure from energy intake. Calculated energy availability values are commonly interpreted as follows: low < 30 kcal/kg FFM/day; moderate 30–45 kcal/kg FFM/day; optimal > 45 kcal/kg FFM/day (Ref. [14]). Energy availability serves as a means to quantify the residual energy available to support an athlete’s physiological functions, and, if below a certain threshold, may be a primary causative factor contributing to RED-S over time [13]. Further, a higher prevalence of LEA is reported in women athletes, as indicated in previous studies [15,16,17,18]. Although valuable, the calculation of energy availability is laborious and can be challenging when working with a large number of athletes. Therefore, screening tools such as the Low Energy Availability in Females Questionnaire (LEAF-Q) [19] have been developed and previously validated in a cohort of women endurance athletes and dancers, but not collegiate team sport athletes. Certain body composition parameters, densitometry metrics, hematological markers, and metabolic tests have also been used to screen for those at risk of RED-S with variable degrees of efficacy [20,21,22]. A better understanding of the utility of these screening tools across multiple populations would help practitioners to identify athletes at risk of LEA.

Common trends among athletes regarding dietary practices indicate a misunderstanding of energy and macronutrient requirements and the role of certain dietary supplements [23], which likely contributes to the dietary deficiencies and occurrence of LEA commonly seen in athletes [24]. However, inconsistencies exist as to how the nutrition knowledge of athletes has been assessed in the past [23]. Therefore, in an effort to create a more standardized method of assessing nutrition knowledge, Trakman et al. [25,26] developed an abridged nutrition knowledge questionnaire that is specific to the dietary requirements of athletes. In theory, a higher level of nutrition knowledge may positively influence an athlete’s dietary behaviors, as previous research in athletes has indicated that those with greater sport nutrition knowledge scores were more likely to self-report healthier dietary practices [27]. Further, previous sport nutrition education interventions have led to marked improvements in nutrition knowledge [4], quality of diet [4,28], body composition, and performance [28] over the course of a season. However, the potential relationship between nutrition knowledge and LEA in athletes has yet to be fully elucidated. 

In the United States, Division III athletes represent ~39% of the National Collegiate Athletic Association, which includes more than 440 Division III women’s soccer programs. Limited data exist in regard to LEA prevalence in collegiate team sport athletes, specifically in women’s soccer. Likewise, the effectiveness of screening tools, such as the LEAF-Q, in identifying those at risk of LEA has yet to be determined, or how nutrition knowledge may influence energy availability in collegiate team sport athletes. Therefore, the purpose of the current study was to examine the prevalence of LEA among a convenience sample of Division III collegiate women soccer athletes and to examine the utility of the LEAF-Q as a tool to screen for those at risk of LEA. A secondary aim was to examine the relationship between nutrition knowledge, energy availability, and dietary intake. 

## 2. Materials and Methods 

### 2.1. Experimental Design

The current observational study began prior to the start of the fall academic term and soccer season, when athletes completed a body composition assessment and an electronic online survey previously developed to assess sport nutrition knowledge. At the soccer season’s midpoint, athletes completed a different online electronic survey to screen for LEA. At this time, athletes also completed a 4-day monitoring period to assess energy availability. Players were asked to record dietary intake and were equipped with a team-based heart rate monitoring system with on-board inertial sensors to assess activity energy expenditure throughout each practice and match during the 4-day monitoring period. 

### 2.2. Subjects

Eighteen National Collegiate Athletic Association (NCAA) Division III women soccer athletes participated in the current study. The participant demographic data are presented in Table 1. All players who were medically cleared were invited to participate in this study. Procedures were approved by the University’s Institutional Review Board for the Protection of Human Subjects (IRB #19-AJ-707). The study was conducted according to the Declaration of Helsinki guidelines. Written consent was obtained from all the subjects prior to data collection. 

### 2.3. Procedures

Upon arrival to the laboratory, height and body mass were recorded to the nearest 0.01 cm and 0.02 kg, respectively, using a stadiometer (Detecto, Webb City, MO, USA) and digital scale (BOD POD model 2000A; BOD POD; Cosmed USA, Concord, CA, USA), with each subject barefoot. Body composition variables (i.e., percent body fat, fat-free mass, and fat mass) were assessed using air displacement plethysmography (BOD POD model 2000A; BOD POD; Cosmed USA, Concord, CA, USA) according to standard operating procedures. The thoracic gas volume was estimated. 

#### 2.3.1. Energy Availability

Athletes were asked to record dietary intake using an online commercially available nutrition analysis program (MyFitnessPal, Under Armour, Baltimore, MD, USA). Prior to this period, they were educated on methods to estimate portion sizes and provided with informational packets and instructional videos to promote accurate self-reporting. Daily energy and macronutrient intakes were averaged across the 4-day monitoring period. Absolute energy and macronutrient intakes (kcal/day or g/day) were recorded and were also made relative to body weight (kcal/kg/day or g/kg/day) to allow for comparison between individuals. Activity energy expenditure was assessed using wearable monitoring devices (Polar TeamPro^TM^ Polar Electro, Oy, Finland) and calculated using proprietary algorithms. Energy availability was then calculated by subtracting the activity energy expenditure from energy intake and expressed as kcals per kilogram of FFM. A threshold of <30 kcal/kg of FFM was used to classify players as having LEA [14].

#### 2.3.2. Low Energy Availability in Females Questionnaire

The LEAF-Q, originally developed as a paper survey, was converted into an online electronic format for ease of distribution and scoring. The 25-item questionnaire asks a series of questions pertaining to prior injury history, gastrointestinal issues, menstrual cycle patterns, and contraception use. A score of ≥8 would classify the athlete as having LEA. It has previously been shown to have an acceptable degree of sensitivity (78%) and specificity (90%) in women athletes and a Cronbach’s alpha ≥ 0.71 [19]. 

#### 2.3.3. Abridged Sport Nutrition Knowledge Questionnaire

The Abridged Sport Nutrition Knowledge Questionnaire (ANSKQ) consists of 37 items that assess general (*n*  =  17) and sport (*n*  =  20) nutrition knowledge and has previously been shown to be a valid and reliable questionnaire and has a PerSepIndex = 0.80 [29]. The scores from the ANSKQ were automatically calculated and categorized using the knowledge scoring system of poor (0–49%), average (50–65%), good (66–75%), and excellent (75–100%) from previously published methods [26].

## 3. Statistical Analysis

A sensitivity and specificity analysis was completed to examine the ability of the LEAF-Q to identify those at risk of LEA. Tests of normality were conducted, and it was found that Shapiro–Wilk was violated for average energy availability (AEA) for non-LEA athletes (*p* = 0.044) and for average energy intake (AEI) for LEA athletes. Thus, differences in AEA and AEI were assessed using the Mann–Whitney U test. Differences in other variables of energy intake between athletes having LEA and athletes without LEA were analyzed using independent samples *t*-tests. Data were considered statistically significant when the probability of a type I error was 0.05 or less. Pearson correlation coefficients were used to examine the relationships between EA, LEAF-Q scores, ASNKQ scores, BF %, FFM, fat mass, body mass, and body mass index. The following criteria were used for interpreting the correlation coefficients: very weak: <0.20; weak: 0.20–0.39; moderate: 0.40–0.59; strong: 0.60–0.79; and very strong: >0.80 [30]. Cohen’s d, utilizing pooled standard deviations, was used to assess the effect sizes for differences in the energy intake variables. The effect sizes were interpreted using the following criteria: 0.2 = trivial; 0.2–0.6 = small; 0.7–1.2 = moderate; 1.3–2.0 = large; and >2.0 = very large. All the data were analyzed using the Statistical Package for the Social Sciences (SPSS, Version 25.0; IBM Corp., Armonk, NY, USA). 

## 4. Results

A total of 66.7% percent of athletes (*n* = 12) presented with LEA (23.0 ± 5.7 kcals/kg FFM) versus non-LEA (*n* = 6; 36.4 ± 7.3 kcals/kg FFM). The LEAF-Q survey tool only identified 56.3% of athletes as at risk of LEA. The sensitivity and specificity analysis yielded a true positive rate of 40.0% and a true negative rate of 16.7% when using the LEAF-Q as a screening tool for LEA. 

Table 2 provides a summary of the dietary intake of all athletes. In comparison to athletes with LEA, athletes without LEA consumed more relative (*p* = 0.004) energy than those with LEA. Athletes without LEA also consumed higher amounts of carbohydrates (absolute: *p* = 0.029; relative: *p* = 0.003) and relative fat (*p* = 0.013) than LEA athletes. No differences were observed between the absolute and relative protein consumed or the absolute fat intake. Additionally, there were no differences in the absolute energy intake between the two groups. 

The mean score from the ASNKQ indicated that 44.7% of the questions were answered correctly. When analyzing the difference in scores between the athletes with and without LEA, the athletes with LEA scored lower compared to the athletes without LEA (40.9 ± 10.4% vs. 52.4 ± 9.8%; *p* = 0.040; ES = 1.14). Moderate inverse relationships were observed between the mean energy availability values and body mass (r = −0.503) and FFM (r = −0.520), and between the ASNKQ scores and fat mass (r = −0.508), as presented in Table 3.

## 5. Discussion

The purpose of the current study was to assess the prevalence of LEA among a cohort of collegiate women soccer athletes and to examine the utility of the LEAF-Q as a tool to screen for those at risk of LEA. A secondary aim was to assess the relationship between nutrition knowledge, energy availability, and energy intake. The main findings of the current study found the prevalence of LEA to be 67% among the current cohort of athletes when assessed directly through dietary analysis and activity energy expenditure. This prevalence rate is higher than previous findings reported in women soccer players at the NCAA Division I (26–33%) [31] and professional levels (23%) [32], as well as in collegiate volleyball (20%) [33] and elite endurance athletes (12–20%) [19]. However, prevalence rates of 40–60% have been observed in collegiate women endurance athletes [15,16], which are only slightly below those observed in the current study. Such similarity was unexpected, considering that, unlike soccer, success in endurance sports requires a high training volume and typically a smaller body type with minimal body fat, which predisposes one to LEA. Therefore, it would be expected that soccer athletes would be less likely to exhibit LEA compared to endurance athletes. The mean energy availability value observed in the current study was 27.5 ± 8.9 kcals/kg FFM/day, which is below the threshold of <30 kcals/kg FFM/day used to classify those with LEA [14], and below that previously reported in women soccer athletes at the professional level (35 kcals/kg FFM/day) [32]. Further, the aforementioned professional soccer athletes [32] had access to nutritional staff, compared to athletes in the current study whose institution did not staff a sport dietitian or nutritionist. 

The high degree of variability in the LEA values across different team sports may be partially attributed to the limited access to sports nutrition education and provisional food (fueling stations), resources that may be more common at more competitive collegiate and elite levels. In fact, previous research has indicated that sport nutrition education interventions and access to a sports dietitian improve eating behaviors and nutritional knowledge in collegiate athletes [4,34,35]. However, some studies have shown [15,36] that, while a nutritional education intervention led to a measurable increase in nutrition knowledge, the prevalence of LEA did not change. Therefore, a combined approach of nutrition education, opportunities to practice dietary skills as well as behavior change therapy [37] may be necessary to help athletes minimize the risk of nutritional deficiencies [38].

The results from the current study indicate that the LEAF-Q was not an effective screening tool, as it only identified 56.3% of the soccer athletes as at risk of LEA, while the direct assessment of LEA identified 66.7% of the athletes as exhibiting LEA. Further, the sensitivity and specificity analysis yielded significantly lower values than those previously published when the tool was validated [19]. Similar findings have also been reported at the professional level [32]; therefore, the LEAF-Q may have limited utility as a screening instrument in women’s soccer, at least when used as a standalone tool. The LEAF-Q has been used successfully in elite sprinters for identifying LEA in conjunction with additional primary indicators, including energy availability, the presence of amenorrhea, low bone mineral density, and hormone abnormalities [39]. Therefore, a comprehensive monitoring plan may be warranted in order to accurately screen athletes and those at risk or in need of further clinical evaluations.

A novel finding from the current study was that athletes with LEA scored lower (41% = poor) on the ANSKQ than athletes without LEA (52% = average) [26], suggesting that lower nutrition knowledge may increase the likelihood of insufficient energy intake, as athletes may have less of an understanding of how to meet the dietary requirements of their sport. Further, moderate differences between those with LEA and those without, as determined by effect size, were observed. Additionally, an inverse relationship was observed between nutrition knowledge and fat mass (r = −0.508), indicating that athletes with lower nutrition knowledge had higher levels of fat mass. There were no additional relationships between nutrition knowledge, energy availability, and body composition values. 

Low energy availability values are one of many metrics that may indicate dietary insufficiencies. The athletes in the current study had a daily mean energy intake of 30 kcals/kg/day, which is below the recommended energy intake of 40–60 kcals/kg for women team sport athletes [7,40] and likely contributed to the high prevalence of LEA observed. Similar findings have been reported in the Under−21 United States Women’s National Soccer team, with an average daily energy intake of 34 kcals/kg/day reported [8]. Additionally, the daily carbohydrate intake of 3.7 g/kg per day observed in the current study was below the recommended 5–12 g/kg of bodyweight for soccer players [41]. This finding is consistent with other studies in women’s soccer, which have frequently noted inadequate carbohydrate intakes ranging from 3.3 to 5.0 g/kg/day [8,9,10,11,12,32]. In the current study, suboptimal energy and macronutrient intakes were more common in athletes with LEA (Table 2). The inability of collegiate athletes to meet nutritional recommendations for their respective sport is not uncommon, as it has been previously reported in both men and women athletes participating in football [42], lacrosse [7], swimming [6], basketball [2], gymnastics [2], and volleyball [33]. While nutritional recommendations have been established for women soccer athletes, the results of the current study demonstrate a continued need for sport nutrition education interventions to be part of regular team activities. Moreover, the high prevalence of LEA is concerning and supports the need to identify individuals at risk of LEA, particularly at lower levels of competition, who may not have access to sport dietitians or other nutrition-centric resources. The early detection of LEA and the implementation of appropriate interventions may reduce the risk of conditions such as RED-S. Therefore, there remains a need for accurate and efficient screening tools to identify those at risk of LEA.

## 6. Limitations

A limitation of the current study is the use of self-reported dietary intake, which may be subject to underreporting by participants [43]. Therefore, there is a potential bias for an overestimation in the prevalence of LEA. Further, the 4-day monitoring period may not be an adequate reflection of the normal dietary habits and activity levels of the athletes throughout the entire season. Therefore, it is possible that, while the LEAF-Q did not accurately identify those at risk of LEA when compared to direct measures of energy availability, the LEAF-Q may be more reflective of long-term energy status, as the questions are directed at health history and symptoms common to RED-S, which may manifest over time. An additional limitation is the small sample size. The prevalence of LEA among the current cohort of women soccer athletes may not be representative of all women soccer athletes across all levels of collegiate competition. 

## 7. Conclusions

The results indicate there may be a high prevalence of LEA among collegiate women soccer athletes. Since the LEAF-Q was not an effective tool in identifying those at risk of LEA, it is recommended that future research examine the utility of LEAF-Q for women athletes participating in team sports. Additionally, the nutrition knowledge of collegiate women soccer athletes was classified as poor-to-average, with the athletes failing to meet several nutritional recommendations for their respective level of training. Athletes with LEA tended to have a lower nutrition knowledge score compared to those without LEA. Therefore, the development of additional screening tools for LEA would be beneficial for the team sport population. Sport nutrition education interventions are recommended to help athletes understand their advanced dietary requirements and provide practical strategies to meet these dietary recommendations. This may help athletes consume adequate amounts of energy and avoid having LEA. 

## Figures and Tables

**Table 1:** Summary of subject demographics (*n* = 18).

Height (m)	1.67 ± 0.1
Body Mass (kg)	65.3 ± 7.9
Fat Free Mass (kg)	49.1 ± 4.7
Body Fat (%)	24.9 ± 5.6
Age (yrs.)	19.2 ± 1.1

Values represented as mean ± standard deviation.

**Table 2:** A summary of average daily energy and macronutrient intake by energy status.

	LEA (*n* = 12)	Non-LEA (*n* = 6)	All (*n* = 18)	Effect Size
Energy Availability (kcals/kg FFM/d)	23.0 ± 5.7	36.4 ± 7.3	27.5 ± 8.9	2.0
Energy Intake (kcal/d)	1806.8 ± 264.0	2179.7 ± 452.0 *	1931.2 ± 371.2	1.0
Relative Energy Intake (kcals/kg/d)	26.9 ± 5.2	36.5 ± 7.0 **	30.1 ± 7.3	1.6
Carbohydrate Intake (g/d)	220.1 ± 36.6	274.3 ± 59.8 *	238.2 ± 51.1	1.1
Relative Carbohydrate intake (g/kg/d)	3.3 ± 0.7	4.6 ± 0.9 **	3.7 ± 1.0	1.6
Protein Intake (g/d)	74.5 ± 17.0	81.1 ± 22.3	76.7 ± 18.5	0.3
Relative Protein Intake (g/kg/d)	1.1 ± 0.3	1.3 ± 0.3	1.2 ± 0.3	0.7
Fat Intake (g/d)	64.1 ± 12.	82.7 ± 26.7	70.3 ± 19.6	0.9
Relative Fat Intake (g/kg/d)	1.0 ± 0.2	1.4 ± 0.4 *	1.1 ± 0.4	1.3

Values represented as mean ± standard deviation. * *p* < 0.05, ** *p* < 0.01.

**Table 3:** Relationships between body composition parameters, energy availability, and sport nutrition knowledge.

	Body Mass (kg)	BF% (%)	FFM (kg)	FM (kg)	Mean EA (kcal/kg FFM)	LEAFQ Score (AU)	ASNKQ (% Correct)
Body Mass (kg)	1	0.479 *	0.774 **	0.828 **	−0.503 *	−0.068	−0.322
BF% (%)		1	−0.135	0.771 **	−0.068	−0.116	−0.183
FFM (kg)			1	0.301	−0.520 *	0.014	−0.066
FM (kg)				1	−0.324	−0.116	−0.508 *
Mean EA (kcal/kg FFM)					1	0.088	0.117
LEAFQ Score (AU)						1	0.410
ASNKQ (% Correct)							1

* Correlation is significant at the 0.05 level (2-tailed). ** Correlation is significant at the 0.01 level (2-tailed). BF% = body fat percentage; FFM = fat-free mass; FM = fat mass; EA = energy availability; LEAFQ = low energy availability in females questionnaire; ASNKQ = abridged sport nutrition knowledge questionnaire.
